# A preliminary study on the quality of street vended foods around a university in Kunming, China

**DOI:** 10.1002/fsn3.1959

**Published:** 2020-10-24

**Authors:** Xuechun Zhang, Yunqian Li, Jian Sun, Huan Kan, Zhenxing Wang, Ping Xiang

**Affiliations:** ^1^ College of Life Sciences Southwest Forestry University Kunming China; ^2^ Agro‐food Science and Technology Research Institute Guangxi Academy of Agricultural Sciences Nanning China; ^3^ Institute of Environmental Remediation and Human Health Southwest Forestry University Kunming China

**Keywords:** composition, food quality, heavy metal, microbiology, oil quality, street vended foods

## Abstract

The street vended foods (SVF) are very popular in China, particularly in highly adolescents populated regions such as schools. Food quality is a critical global issue, but there are few studies describe the quality assessment of SVF. In this study, the quality of SVF around a university in Kunming was evaluated, including the microbial quality, proximate composition, oil quality, and heavy metal content. Microbial results showed that the aerobic plate count (APC) and *Escherichia coli* (*E. coli*) counts ranged from 1.94 to 7.43 log CFU/g or ml, and 0.53 to 1.48 log CFU/g or ml, respectively. A portion of fried snack samples exceed the standard limit of acid value (AV) and peroxide value (POV), and the same result was observed in carbonyl group value (CGV), thiobarbituric acid (TBA) value, viscosity, and conductivity. The proximate composition of the fried snack samples varied widely, while the fat content was generally higher. The heavy metal analysis showed most samples met the safety standards, with the content of 12–51, 1–19, 12–73, and 11–88 μg/kg for As, Hg, Cd, and Pb, respectively. In conclusion, although the overall results of this study were satisfying, more attention should be given to the quality of SVF. Consequently, there is a need for additional measures to protect consumers, particularly young adults in college, from foodborne disease.

## Graphical abstract

The microbial quality, proximate composition, oil quality, and heavy metal content of street vended foods around a university in Kunming was evaluated. In conclusion, although the overall results of this study were satisfying, more attention should be given to the quality of street vended foods.

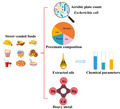

## 1 INTRODUCTION

Street vended foods (SVF) is classified as food and beverages prepared and sold on streets and in public places for immediate consumption World Health Organization. Food Safety, T., (1996). Due to its convenience, economy, and pungent flavor, SVF gain a lot of popularity among consumers worldwide, more than 150,000 kinds of SVF are being consumed in China alone (Lin et al., 2017).

However, most SVF are prepared in outdoor conditions and commonly encountered with the poor hygiene, improper sanitation, and lack of clean water (Ryu et al., 2011). During the preparation and distribution process of SVF, there is a high risk of foodborne disease, because of the questionable quality of raw materials, poor environment, infrastructure, preparation, and the personal hygiene of the food handlers (Islam et al., 2015; Mepba et al., 2007). Previous studies had shown that some SVF exceeded the local total plate count and total coliforms standards in some countries (Manguiat & Fang, 2013; Noor, 2016), and some pathogenic bacteria were found (Manguiat & Fang, 2013). In addition, fried, grilled, and barbecued foods are the most frequently consumed SVF in recent years. A variety of chemical reactions (hydrolysis, oxidation, and polymerization) occur while oil high‐temperature cooking, lead to poor quality of oil, these raise more considerable attention (Rose et al., 2015). It has been reported that the polar compound, chemical, and physical properties of oils from some SVF did not meet certification standards (Bou et al., 2012). On the other hand, the consumption of SVF was closely related to the intake of fats, saturated fatty acids, and trans‐fatty acid, as well as the occurrence of obesity and noncommunicable diseases (Steyn et al., 2014). Besides, the heavy metals concentration in some SVF exceeded the tolerable daily intake (Hariri et al., 2015). Simultaneously, the quality issues including nonpermitted colors, artificial sweeteners, adulterant oils, and poor sanitary quality were found in some SVF (Chandrasekhar et al., 2003).

The quality of SVF is a critical issue in densely populated areas such as university campuses, many college students tend to consume the SVF and are easy to buy from retail or mobile stores near the campus (Kim et al., 2013; Nyenje et al., 2012). In China, there are 2,956 universities, and more than 28 million college students (Statistics, N. B. o, 2020). The consumption of SVF in Chinese college students is very considerable and increasing constantly. Consequently, it is necessary to conduct a systematic evaluation of SVF quality around Chinese university campuses. Although some studies have assessed the microbial quality of SVF, the available information on its microbial contamination and oil quality is still little.

Based on the potential hazards of the SVF, this paper analyzed the physical, chemical, and microbial qualities of SVF near a university in Kunming city, China. Our work can provide some valuable suggestions for the dietary reference of college students and could be useful for authorities to improve SVF management strategies, and develop sanitation rules.

## 2 MATERIALS AND METHODS

### 2.1 Samples collection

The SVF samples were collected from the fixed and floating stalls around a university located in Kunming city, China, from September to October 2019. In brief, 133 different SVF samples (about 200 g/each sample) were randomly collected, comprising salads, pasta, soy products, fries, and grilled or baked snacks. The samples were wrapped in sterile polyethylene bags and immediately transported to the refrigerator (4°C). The microbial quality, oil quality, proximate composition, and heavy metal content of samples were analyzed within 24 h.

### 2.2 Microbiological measurement

According to the types of preparing methods, the samples were subdivided into 5 groups namely *fully cooked food*, *fully cooked food with minimum handling prior to consumption*, *multi‐ingredients preparations*, *raw vegetables* (*fruits) ready for consumption*, and *Handmade drinks* (Table 1). The aerobic plate count (APC) and *Escherichia coli* (*E. coli*) count were enumerated according to the procedures described by Ng et al. (2013). In brief, samples (25 g) were cut into pieces with sterile scissors, then 225 ml of physiological saline solutions were added and made a ten‐fold dilution, then the suspensions were homogenized using a high shear homogenizer (Dalong, China) at 2,810 *g* for 1 min. The 100× dilutions were obtained using the same method. (a) *APC count*: 1 ml of dilutions were added in soybean casein agar medium, after incubation at 37°C for 1–2 days, the colonies between 30 and 300 of the plates were counted. (b) *E. coli count*: 1 ml diluted liquid was plated and incubated at 32°C for 2 days on *E. coli* count Petri film plates (3 M) under aerobic conditions. After the completion of incubation, plates were counted on a standard colony counter. The number of APC and *E. coli* were converted to log CFU/g or ml.

**TABLE 1:** Quantitative contamination levels of aerobic plate count (APC) and *Escherichia coli* (*E. coli*) in food samples

Group	Food type	Samples	Microbial population (CFU/g or ml)	
			APC^d^	*E. coli* ^e^
1	Fully cooked food^a^	Pancakes, French fries	<10^5^	<100
2	Fully cooked food with minimum further handling prior to consumption	Chinese burgers, fermented Tofu	<10^6^	<100
3	Multi‐ingredients preparations^b^	Cold dishes with dressing, mixed salads, sushi	<10^7^	<100
4	Raw vegetables and fruits ready for consumption	Cucumber in sauce, seaweed salad	N/A^c^	<100
5	Handmade drinks	Tofu pudding, soybean milk	<10^4^	<100

^a^ This food was eaten or sold immediately.

^b^ Fully cooked foods with further handling like slicing, mixing and refrigeration prior to consumption.

^c^ Not applicable.

^d^ Aerobic plate count.

^e^ *Escherichia coli*.

### 2.3 Proximate composition analysis

The moisture, fat, protein, and ash of samples were analyzed using the standard procedures of the AOAC (2000).

### 2.4 Chemical parameter analyses of oils

The lipid samples were extracted with petroleum ether (boiling point 30–60°C) and dried using a rotary evaporator at 40°C for further analyses (Chen et al., 2018). The acid value (AV) and peroxide value (POV) were determined according to AOCS and expressed as mg/g sample (AOCS, 2017). The carbonyl group value (CGV) was determined by the dinitrophenyl hydrazine (DNPH) method and expressed as mmol/g sample (Endo et al., 2001). The thiobarbituric acid (TBA) value was determined according to the previous method and expressed as mg malonaldehyde (MDA)/kg sample (Chen et al., 2018). The inhibitions of the antioxidant activity were measured according to the published method (Kamel & El Sheikh, 2012).

### 2.5 Analyses of color index, viscosity, and conductivity of oil

The color indexes of extracted oils were determined by measuring the absorbance of 2.5% oil (oil in isooctane, w/v) at 470 nm (Yoshida & Kajimoto, 1989). The color parameters including L^*^, a^*^, b^*^ value of oil were measured using a colorimeter (Beituo SC‐80) according to the previous report (Krokida et al., 2001). The viscosity was measured at room temperature using a rotary viscosimeter (Jingke DNJ). Conductivity was determined according to the report of Li et al. (2016). In brief, 20 g extracted oil was mixed with 50 ml deionized water in a separating funnel (250 ml) at room temperature, the mixtures were stirred for approximately 5 s and held for 5 min, then conductivity was measured using the conductor (Shengxi DDSJ‐308A EC).

### 2.6 Heavy metal analyses of the food samples

The heavy metal (Cd and Pb) contents were determined by a graphite furnace atomic absorption spectrophotometer (Pu Analysis TAS‐990) with Zeeman background correction (Ay & Karayunlu, 2008). In addition, the inductively coupled plasma mass spectrometry (ICP‐MS) (Thermo Scientific) was used to measure the content of Hg and As (Yim et al., 2017).

### 2.7 Statistical analysis

The analysis was carried out in triplicates for all determinations and the results of the triplicate were expressed as mean ± standard deviation (*SD*).

## 3 RESULTS

### 3.1 Microbial analysis of SVF samples

**FIGURE 1:**
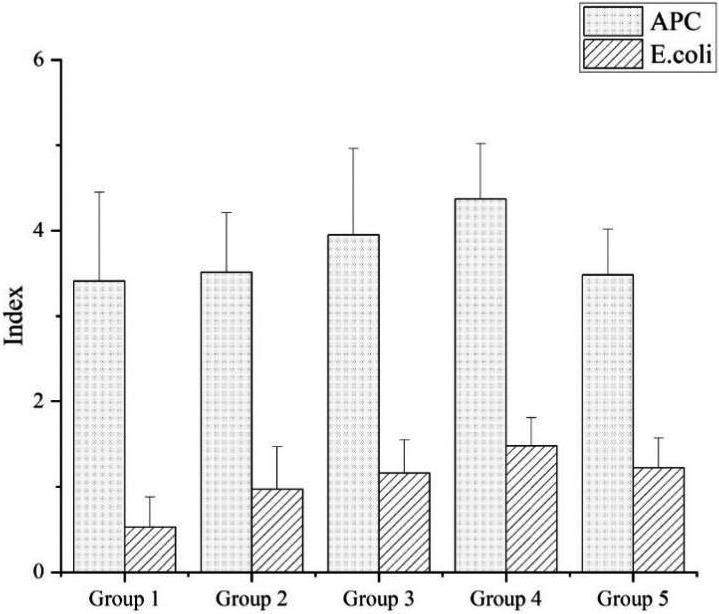
The aerobic plate count (APC) and *Escherichia coli* (*E. coli*) of street vended foods samples. Data are expressed as mean (log CFU/g or ml) ± standard error of the mean. Group 1, fully cooked food; Group 2, fully cooked food with minimum further handling prior to consumption; Group 3, multi‐ingredients preparations; Group 4, raw vegetables and fruits ready for consumption; Group 5, handmade drinks

Figure 1 showed the numbers of APC and *E. coli* of SVF. The results indicated that the average APC value of tested 5 group samples ranged from 3.41 to 4.37 log CFU/g or ml. According to the microbial standards set by the Chinese Population and Family Planning Commission, 13 of 74 samples (17.6%) were out of limits of APC. The highest exceedance was category **3** (*multi‐ingredients preparations*), the lowest was category **1** (*fully cooked food*). The highest numbers of APC were found in category **4** (*raw vegetables and fruits ready for consumption*), whereas category **1** (Table 2).

**TABLE 2:** Incidence and levels of aerobic plate count (APC) in the food samples

Groups^a^	*n*	Sample (%) meet standards	Percentage (%) of samples in the indicated intervals (log CFU/g or ml)				Range (log CFU/g or ml)	Mean (log CFU/g or ml)
			<3	3–4	4–5	>5		
1	14	92.9	21.4	57.1	14.3	7.2	2.20–5.56	3.41 ± 2.07
2	22	81.8	9.1	40.9	36.4	13.6	1.94–6.72	3.51 ± 1.40
3	22	77.3	4.5	31.8	45.5	18.2	2.41–7.43	3.95 ± 2.01
4	22	N/A	4.5	18.2	40.9	36.4	3.38–6.91	4.37 ± 1.29
5	16	81.3	12.5	56.3	25.0	6.2	2.64–5.99	3.48 ± 1.08

^a^ Group 1, fully cooked food, Group 2, fully cooked food with minimum handling prior to consumption, Group 3, multi‐ingredients preparations, Group 4, raw vegetables and fruits ready for consumption, Group 5, handmade drinks.

The average number of *E. coli* ranged from 0.53 to 1.48 log CFU/g or ml (Figure 1), and the highest numbers of *E. coli* were observed at category **4**, whereas category **1**. According to the *E. coli* count standard from the Chinese Population (100 CFU/g or ml), 10 of 96 samples were out of limits of *E. coli*. The exceedance of *E. coli* from high to low is category **3**, **4**, **2**, **5**, and **1** (Table 3).

**TABLE 3:** Incidence and levels of *Escherichia coli* (*E. coli*) in the food samples

Groups^a^	*n*	Sample (%) meet standards	Percentage (%) of samples in the indicated intervals (log CFU/g or ml)				Range (log CFU/g or ml)	Mean (log CFU/g or ml)
			<3	3–4	4–5	>5		
1	14	92.9	14.3	50.0	28.6	7.1	0–2.04	0.53 ± 0.35
2	22	95.5	9.1	45.5	40.9	4.5	0–2.08	0.97 ± 0.50
3	22	86.4	4.5	31.9	50.0	13.6	0–3.08	1.16 ± 0.78
4	22	81.8	4.5	22.8	54.5	18.2	0–2.26	1.48 ± 0.66
5	16	93.8	12.5	25.0	56.3	6.2	0–2.12	1.22 ± 0.35

^a^ Group 1, fully cooked food, Group 2, fully cooked food with minimum handling prior to consumption, Group 3, multi‐ingredients preparations, Group 4, raw vegetables and fruits ready for consumption, Group 5, handmade drinks.

### 3.2 Evaluation of oil qualities

As showed in Table 4, among all the fried snack samples, AV of fried fermented Tofu (1.75 mg/g) was the highest, while pancake was the lowest (0.75 mg/g). According to the statistical results, 6.8% (9/133) fried food samples exceeded the AV standard, and the French fries showed the highest exceedance. These may be due to the fact that oils used in French fries tend to be frying temperature and reused frequently. 8.3% (11/133) fried food samples exceeded the POV standard, and barbecues showed the highest POV (14.97 meq/kg) and exceedance. The CGV (22.6 meq/kg) of fried steamed buns was highest in tested fried samples, and 10.5% (14/133) samples were out of limit, barbecue showed the largest exceedance. The French fries showed the highest TBA value (0.86 mg/kg) in tested samples, and 10.5% (2/19) French fries samples exceeded 1 mg/kg MDA.

**TABLE 4:** Quality parameters of lipid extracts obtained from fried snack samples

Samples	*n*	AV	ESR of AV	POV	ESR of POV	CGV	ESR of CGV	TBA value	ESR of TBA^a^
		mg/g	%	meq/kg	%	meq/kg	%	mg/kg	%
Fried rice noodles	20	1.07 ± 0.12	0	8.67 ± 0.79	0	12.8 ± 0.5	0	0.29 ± 0.01	0
Fried drumstick	12	1.21 ± 0.06	8.3	12.61 ± 0.76	8.3	10.4 ± 1.0	16.7	0.41 ± 0.00	0
Pancakes	18	0.75 ± 0.09	5.6	10.24 ± 0.51	5.6	9.7 ± 1.0	5.6	0.38 ± 0.00	0
Fried steamed buns	7	1.19 ± 0.14	14.3	14.18 ± 1.55	28.6	22.6 ± 2.0	28.6	0.81 ± 0.01	14.3
Roasted rice cake	12	0.83 ± 0.07	0	3.94 ± 0.62	0	5.1 ± 0.4	0	0.26 ± 0.01	0
Fried rice	15	0.92 ± 0.09	0	10.24 ± 0.88	0	11.1 ± 0.6	0	0.25 ± 0.00	0
Fried fermented Tofu	16	1.75 ± 0.17	6.3	12.61 ± 2.84	12.5	12.8 ± 3.0	12.5	0.69 ± 0.03	6.3
Barbecue	14	1.35 ± 0.12	14.3	14.97 ± 1.56	21.4	14.6 ± 0.4	28.6	0.58 ± 0.00	0
French fries	19	1.25 ± 0.22	15.8	12.61 ± 1.42	10.5	16.6 ± 0.3	15.8	0.86 ± 0.03	10.5

Abbreviations: AV, acid value; CGV, carbonyl group value; ESR, Exceeding standard rate; POV, peroxide value; TBA, thiobarbituric Acid.

^a^ Exceeding standard rate over 1 mg/kg.

**FIGURE 2:**
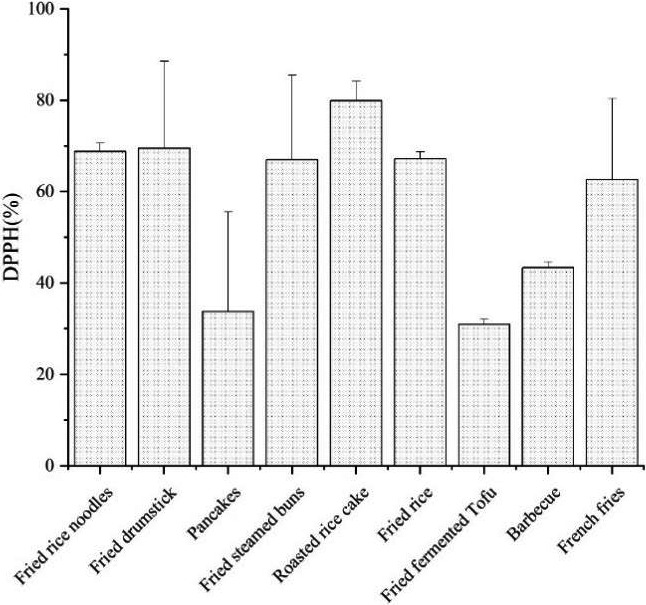
Total antioxidant activity of the oils extracted from fried snack samples

Figure 2 presents the antioxidant activity of SVF samples. As showed in the figure, antioxidant activity of fried fermented Tofu was the lowest (31.0%), this may due to the high cooking temperature of frying destroyed the polyphenols in oil resulting in reduced antioxidant capacity.

From Table 5, the absorbance value at 470 nm of oils extracted from SVF samples ranged from 0.189 to 0.422. Among the fried snack samples, barbecue showed the highest absorption value while fried rice showed the lowest value. Correspondingly, L* value (30.29) of barbecue was the lowest, a* value (18.97) of fried drumstick and b* value (33.20) of fried fermented Tofu were the highest, these results indicated that the color of the barbecue, fried drumstick, and fried fermented Tofu had darkened by nonenzymatic browning during the cooking process.

**TABLE 5:** Color index, apparent viscosity, and conductivity of lipid extracts obtained from fried snack samples

Samples	*n*	Absorption	Chroma value			Viscosity	Conductivity
		At 470 nm	L*	a*	b*	mPa s	μs/cm
Fried rice noodles	20	0.288	67.74 ± 0.02	13.30 ± 0.67	17.39 ± 0.42	52.5 ± 1.4	12.54 ± 0.06
Fried drumstick	12	0.329	52.73 ± 0.05	18.97 ± 0.12	14.60 ± 0.28	60.9 ± 2.9	12.51 ± 0.04
Pancakes	18	0.319	55.78 ± 0.12	3.15 ± 0.24	10.80 ± 0.54	53.2 ± 1.2	11.93 ± 0.07
Fried steamed buns	7	0.369	40.77 ± 0.10	11.99 ± 1.01	26.56 ± 0.67	66.4 ± 1.3	14.34 ± 0.08
Roasted rice cake	12	0.198	67.62 ± 1.17	7.82 ± 0.18	19.57 ± 0.36	53.7 ± 0.7	12.21 ± 0.07
Fried rice	15	0.189	72.36 ± 2.71	1.63 ± 0.07	21.00 ± 0.55	51.0 ± 1.6	14.44 ± 0.09
Fried fermented Tofu	16	0.333	62.69 ± 0.62	5.43 ± 0.05	33.20 ± 0.47	68.6 ± 2.4	24.90 ± 0.11
Barbecue	14	0.422	30.29 ± 0.45	12.25 ± 0.06	12.00 ± 0.26	55.3 ± 2.5	17.09 ± 0.06
French fries	19	0.318	62.34 ± 0.98	−3.38 ± 0.05	13.15 ± 0.44	71.0 ± 1.8	26.28 ± 0.10

The viscosity of oils extracted from SVF samples ranged from 51.0 to 71.0 mPa s, and the French fries showed the highest viscosity. The viscosity result was corresponding to the results of acid value. As shown in Table 5, conductivity of oils ranged from 11.93 to 26.28 μs/cm, and French fries had the highest conductivity, these findings were corresponding to the results of acid value and peroxide value.

### 3.3 Evaluation of proximate composition

The proximate composition of SVF samples was shown in Table 6. The moisture content ranged from 9.22% to 50.61%. The moisture content of fried drumstick and fried rice noodles were significantly higher than other samples, and the French fries was the lowest value. The difference of water content was mainly caused by the different water content of raw materials and the different preparation methods. Therefore, the low moisture content of French fries may cause by the moisture losing during deep frying. The ash contents of SVF ranged from 0.40% to 1.82%. Among the SVF, ash contents of fried fermented Tofu and barbecue were higher than other samples, which may be due to the rich mineral content of soybean and meat. The fat content of tested samples varied from 10.32% to 47.30%. The fat content of French fries was significantly higher than other samples, mainly due to potatoes absorb more oil during deep frying. In general, fat content of fried food samples is relatively high (>10%). With respect to protein content, the fried fermented Tofu and fried drumstick were rich in protein, while the fried rice and rice noodles showed the lowest protein content, which was determined by the composition of the raw materials. In other words, the higher level of bean and meat, the higher protein content of SVF.

**TABLE 6:** Proximate composition of street vended food samples

Samples	*n*	Moisture (%)	Ash (%)	Fat (%)	Protein (%)
Fried rice noodles	20	51.20 ± 1.44	0.74 ± 0.12	10.32 ± 0.83	3.02 ± 0.65
Fried drumstick	12	50.61 ± 2.49	1.57 ± 0.28	16.83 ± 1.24	22.63 ± 3.23
Pancakes	18	23.32 ± 1.02	1.32 ± 0.25	16.51 ± 2.65	8.79 ± 1.15
Fried steamed buns	7	29.85 ± 2.14	0.40 ± 0.07	18.20 ± 1.49	7.83 ± 0.95
Roasted rice cake	12	41.93 ± 3.23	0.84 ± 0.13	11.34 ± 0.68	5.30 ± 0.87
Fried rice	15	43.13 ± 1.76	0.63 ± 0.11	10.41 ± 1.12	3.16 ± 0.37
Fried fermented Tofu	16	20.87 ± 2.96	1.82 ± 0.18	14.81 ± 2.07	32.76 ± 3.58
Barbecue	14	47.51 ± 3.04	1.71 + 0.04	10.71 ± 0.73	18.36 ± 2.26
French fries	19	9.22 ± 1.94	1.13 ± 0.27	47.30 ± 2.69	4.10 ± 0.34

### 3.4 Evaluation of heavy metal content

The four heavy metals contents of selected SVF samples were presented in Table 7. The levels of arsenic ranged from 0.012 to 0.051 mg/kg. The highest and lowest levels of arsenic were found in barbecue and French fries, respectively. The permitted limit of arsenic is 0.5 mg/kg set by the National Health and Family Planning Commission (NPFPC) (2017). As showed in Table 7, all of the samples met the arsenic permitted standard.

**TABLE 7:** Content of heavy metals of street vended foods

Samples	As (mg/kg)			Hg (mg/kg)			Cd (mg/kg)			Pb (mg/kg)		
	Mean	Min.	Max.	Mean	Min.	Max.	Mean	Min.	Max.	Mean	Min.	Max.
Fried rice noodles	0.046 ± 0.012	0.005	0.120	0.005 ± 0.003	0.003	0.013	0.018 ± 0.011	0.001	0.080	0.022 ± 0.018	0.003	0.060
Fried drumstick	0.014 ± 0.008	0.010	0.250	0.019 ± 0.005	–	0.033	0.026 ± 0.015	0.005	0.072	0.062 ± 0.031	0.002	0.660
Pancakes	0.032 ± 0.016	0.003	0.196	0.003 ± 0.001	–	0.013	0.019 ± 0.008	–	0.068	0.014 ± 0.012	0.002	0.160
Fried steamed buns	0.023 ± 0.010	0.003	0.107	0.001 ± 0.001	–	0.019	0.014 ± 0.010	–	0.070	0.011 ± 0.008	–	0.168
Roasted rice cake	0.029 ± 0.013	0.020	0.060	0.001 ± 0.001	–	0.004	0.015 ± 0.006	0.001	0.092	0.015 ± 0.006	0.002	0.069
Fried rice	0.036 ± 0.019	0.003	0.048	0.001 ± 0.001	–	0.027	0.022 ± 0.014	0.001	0.080	0.017 ± 0.008	0.003	0.023
Fried fermented Tofu	0.016 ± 0.011	–^a^	0.370	–	–	0.009	0.073 ± 0.042	0.001	0.380	0.054 ± 0.022	–	0.563
Barbecue	0.051 ± 0.024	–	0.091	0.004 ± 0.001	–	0.057	0.028 ± 0.017	–	0.038	0.088 ± 0.061	0.056	0.127
French fries	0.012 ± 0.006	0.005	0.068	0.001 ± 0.001	–	0.017	0.012 ± 0.007	0.003	0.049	0.060 ± 0.034	–	0.42

^a^ Not detected.

In terms of mercury content, fried drumstick had the highest mercury content (0.019 mg/kg), while fried fermented Tofu had the lowest mercury content (ND) (Table 7), which may be due to the difference in Hg content in the recipes. Such as 1 of 15 (6.7%) tested fried rice samples, and 1 of 14 (7.1%) barbecue exceeded the standard set by the NPFPC (2017) (Table 8).

**TABLE 8:** Sample size and rate of over permitted standard of four heavy metals

Samples	*n*	Rate of over permitted standard (%)			
		As	Hg	Cd	Pb
Fried rice noodles	20	0	0	0	0
Fried drumstick	12	0	0	0	8.3
Pancakes	18	0	0	0	0
Fried steamed buns	7	0	0	0	0
Roasted rice cake	12	0	0	0	0
Fried rice	15	0	6.7	0	0
Fried fermented Tofu	16	0	0	6.3	6.3
Barbecue	14	0	7.1	0	0
French fries	19	0	0	0	5.3

Among all samples, the Cd level of fried fermented Tofu was the highest (0.073 mg/kg) and French fries was the lowest (0.012 mg/kg) (Table 7). According to standards set by the Ministry of Health of China, Cd content in grain, peanut, and meat products should not exceed 0.1 mg/kg, and soybean products should not exceed 0.2 mg/kg. The Cd content of 1 of 16 (6.3%) fried fermented Tofu samples exceeded the standard (Table 8).

The lead content in the tested samples ranged from 0.011 to 0.088 mg/kg (Table 7). Among them, barbecue had the highest lead content, the fried steam bun had the lowest lead content. The maximum permissible limit of lead in grain, soybean, and meat products is 0.5 mg/kg, and the Pb content of 1 of 12 (8.3%) fried drumstick, 1 of 16 (6.3%) fried fermented Tofu, and 1 of 19 (5.3%) French fries exceed the permitted limit (Table 8), which may be related to soil pollution in food‐growing fields, and abuse of the sewage sludge, fertilizers, and pesticides.

## 4 DISCUSSION

### 4.1 Microbial analysis of SVF samples

Generally, the APC is used to evaluate the hygienic quality and bacterial contamination in foods which can reflect the total hygienic quality of SVF, the *E. coli* can be used to represent the fecal pollution. If *E. coli* was detected in foods, it may indicate that there were also other pathogenic intestinal flora existed, such as salmonella (Metz et al., 2020).

APC results suggested that some SVF like category **4**, **3**, and **2** were susceptible to bacterial contamination, which may be caused by sterilization instruction, handing methods, preserving conditions, personal hygiene of counter‐tops (Sibanyoni & Tab it, 2019). Meanwhile, the microbial population was affected by the temporal relationship between food preparation and service (Marzano & Balzaretti, 2013). In addition, the unheated foods such as salad were generally served raw and naturally contaminated may contain high levels of microorganisms that can cause excessive levels of APC and *E. coli*.

In the present study, APC numbers of tested SVF samples were near those reported in Korean elementary schools, which indicated that 15% of nonheated foods and 9% of heated/nonheated food samples were not meet food standards (Ryu et al., 2011).

SVF in China is characterized by large scale, low threshold, and high risk of cross‐contamination. Therefore, it is necessary to supervise the control microbiology condition of SVF by the local government. Some of the SVF did not comply with the regulations governing, and general hygiene requirements for food, the competent authorities should analyze the cause of noncompliance and take immediate remedial action to reduce the amount APC and *E. coli* in food to a satisfactory level (Sibanyoni & Tab it, 2019).

### 4.2 Evaluation of oil qualities

The acid value is the rancidity index of oils, which related to the quality evaluation and of conserving the method of oil. Peroxides, intermediate products, are produced during lipid oxidation, and easily decomposed into volatile and nonvolatile fatty acids, aldehydes, ketones, etc. The peroxide value can be used to characterize the hydroperoxides formed during oils frying/baking. In addition, carbonyl group value is often used as the index of peroxide content and rancidity degree and TBA value could express as the degree of lipid peroxidation products which is used as the index of lipid peroxidation.

A portion of quality parameters of lipid extracts obtained from SVF exceeded the standards set by Chinese national standards Health (SAC, 2003), these may be attributed to the partial dehydration of food materials and the increasing oxidation of unsaturated fatty acids at the relatively high temperature of cooking or frying (Burgos‐Edwards et al., 2017).

The DPPH scavenging capacities of oils is related with the total phenolic contents and cooking conditions, some cooking oils and spices contain the high level of antioxidants, which can exhibit the strong DPPH free radical scavenging capacity (antioxidant ability). Some SVF such as fried fermented Tofu showed low DPPH scavenging capacities (Figure 2), may be attributed to the high‐temperature cooking methods, especially frying, roasting, and grilling (Kobylinski et al., 2016).

During high temperatures and prolonged heating, the L* value of oil usually decreased with the increasing temperature of frying oil due to the water decrease, oil migration, and Maillard reaction (Kumar et al., 2017). Besides, the pigments of sauces added in foods, as well as the pigments existing in used plant oils, such as canola oil, soybean oil, and palm oil (Sarikaya et al., 2016) decreased the lightness, while increased the redness and yellowness of the oil.

Oil viscosity usually has a good correlation with the percentage of free fatty acids and polymers (Safari et al., 2018), the higher acid value, the larger oil viscosity (Tables 5 and 6), which may be due to the formation and accumulation of undesirable components when the oil exposed to oxygen at high temperature (Kumar et al., 2017).

As reported by Yang et al. (2016), the conductivity of cooking oils were greatly affected by the volatile decomposition products and impurities, such as aldehydes, ketones, and alcohols, which mainly contributed to the conductivity of oils. Oils extracted from some SVF showed relatively high conductivity (Table 6) mainly due to the long cooking time and high frying frequency (Yang et al., 2016).

### 4.3 Evaluation of proximate composition

The difference of proximate composition of SVF was mainly caused by the different nutrient content of raw materials and the different preparation methods (Durazzo et al., 2019). In general, fat content of fried food samples is relatively high (>10%). Therefore, it is not conducive to human health with large amount intakes to fried foods. All in all, nutrients of SVF varied widely because of the recipe formulation and cooking methods, and but the fat content was generally higher.

### 4.4 Evaluation of heavy metal content

Heavy mental including arsenic, mercury, cadmium, and lead are toxic elements and often harmful to human body if their level exceeds maximum permitted value. For instance, arsenic is cytotoxic to many organs in the human body and usually causes neurasthenia, multiple skin lesions, and neuritis. Mercury can cause stomatitis and neuropsychiatric symptoms. Cadmium can accumulate in the kidneys and liver and cause cancer. Long‐term consumption of leaded foods could cause serious damage to the blood and nervous systems, especially to children's health and intelligence.

Arsenic content in most SVF samples was relatively low, such as lower than 0.024 mg/kg in meat, 0.103 mg/kg in rice, and 0.036 mg/kg in beans (Li et al., 2011). With respect to mercury, Fried drumstick contain more Hg (Table 7) than meat products in Chile, and the Hg content (<0.001 mg/kg) of fried fermented Tofu was similar to that of boiled beans in Italia faculty cafeteria (Alberti‐Fidanza et al., 2002). In terms of cadmium content, the averaged Cd content of fried fermented Tofu was a little higher than pulses consumed in Italy faculty cafeteria (0.04 mg/kg) (Alberti‐Fidanza et al., 2002). In addition, the Cd content of fried drumstick and French fries were remarkably lower than the meat consumed in Italy faculty cafeteria (0.013 mg/kg) and potato chips sold in Turkey (0.64 mg/kg) (Alberti‐Fidanza et al., 2002; Hariri et al., 2015). Furthermore, the Pb levels of tested barbecue were close to the pork belly consumed in Korean (0.08 mg/kg) (Ryu et al., 2011).

In the light of our researches, the levels of heavy metals in the SVF tested were mostly in line with Ministry of Health of China. The heavy metal level for SVF samples around the university was agreed with the literature reported for the same kind of samples around the country.

## 5 CONCLUSIONS

A certain proportion of samples did not meet the APC and *E. coli* standards, suggesting that the conditions for the preservation, processing, and hygiene of SVF should be improved. The local government should pay attention to the administration of street stall, and develop unified standards of whole restaurant foods. Some SVF contain too much fat, and qualities like AV, POV, and CGV of some oil were not satisfied with standards. In terms of heavy metal levels, some SVF exceeded the standards, but most analyzed samples had low‐level content. The relative inconsistencies in the microorganism's contents and fats quality should be given due attention. Therefore, a balanced diet is highly recommended, such as reducing consumption of SVF around the university. For SVF producers, more efforts should be made to improve the sanitary conditions and awareness of food safety. Eventually, better management strategies and hygiene rules should be developed.

## CONFLICT OF INTEREST

No conflict of interest was declared by the authors.

## ETHICAL APPROVAL

This study does not involve any human or animal testing.
